# Risk prediction for liver injury in Epstein-Barr virus infection in pediatric respiratory tract infections

**DOI:** 10.1186/s13052-023-01546-0

**Published:** 2023-10-11

**Authors:** Song Mao, Liangxia Wu, Wenjing Shi

**Affiliations:** Department of Pediatrics, Shanghai Sixth People’s Hospital, Affiliated to Shanghai Jiao Tong University School of Medicine, Shanghai, China

**Keywords:** Epstein-Barr virus, Liver injury, Infection, Children

## Abstract

**Background:**

Epstein-Barr virus (EBV) infection is likely to co-occur in pediatric respiratory tract infections (RTIs). Liver injury is the common complication of EBV infection. The detailed risk factors for liver injury in EBV infection remain elusive. We aimed to investigate the incidence, characteristics and potential risk factors for liver injury in EBV infection for early risk prediction.

**Methods:**

We retrospectively recruited the pediatric RTIs cases with EBV infection according to a predefined criteria from our hospital between January 2015 and December 2017. We extracted the clinical and laboratory data from the electronical medical records. The impact of age, gender, and various parameters on the liver injury risk was investigated. Univariate logistic regression analysis was performed to analyse the association between clinical/laboratory parameters and liver injury. The related indexes were enrolled in the multivariate logistic regression analysis. Decision curve analysis was used to yield the value of related parameters in predicting liver injury. Receiver operating curve (ROC) analysis was applied to produce the C-index of white blood cell (WBC) count for liver injury. We also tested the non-linear association between WBC count and alanine aminotransferase (ALT).

**Results:**

A total of 216 pediatric RTIs with EBV infection were enrolled. EBV infection is more likely to occur during the winter season. Cytomegalovirus infection was independently associated with liver injury in EBV infection (OR = 6.972, 95% CI = 1.648–29.490, *p* = 0.008). WBC count was independently associated with liver injury in EBV infection (OR = 1.169, 95% CI = 1.051–1.301, *p* = 0.004). The P interaction value between WBC count and cytomegalovirus was 0.149. The decision curve analysis showed that WBC count had larger area under curve compared with platelet (PLT) and birthweight (BW). ROC analysis yielded the c-index of WBC count: 0.75 and cut-point of 8.3. The turning point of WBC count in its association with ALT was 16.8. The *p* value before and after the turning point was < 0.001 and 0.123, respectively.

**Conclusions:**

Cytomegalovirus co-infection demonstrated 5.972 more times of liver injury risk in EBV infection. WBC count was an independent biomarker for liver injury before the turning point of 16.8 in EBV infection. More attention should be paid to the risk of EBV infection in the winter. Cytomegalovirus infection and WBC count merit attention in the monitoring of possible liver injury in EBV infection among pediatric RTIs.

## Introduction

Epstein-Barr virus (EBV), a kind of herpesvirus, is likely to lead to the onset of infectious mononucleosis presenting mainly with fever, oropharyngitis, lymphadenitis, and even acute hepatitis [1]. Meanwhile, EBV coinfection often occurs in various respiratory tract infections (RTIs) in children without typical symptoms. The EBV spreads mainly through the close contact, such as droplet transmission [2]. The incidence of EBV is high among children in the kindergarten due to the small area activities, particularly during the epidemic season. Although EBV itself is a self-limited disease, some cases are prone to be complicated with other dysregulations, even progressing to liver failure [3]. EBV infection is also an important cause of nasopharyngeal carcinoma and lymphomas [4]. Hence, a comprehensive understanding of EBV infection in children with RTIs seems imperative.

EBV infection may lead to significant morbidity and mortality in children with low immunity. EBV is considered as the risk factor for some immunological disorders, such as multiple sclerosis [5]. EBV infection is also likely to induce renal injury and inflammation [6]. Among the EBV- induced complications, liver injury is a very common symptom, hepatic involvement accounts for 80–90% of EBV infection [7]. Severe EBV cases may be complicated with hepatitis [8]. Although some liver injury cases were self-limited, immunocompromised and immunocompetent cases can develop severe, or even fatal acute liver injury [9]. A small number of severe EBV infection cases may be complicated with chronic hepatitis, liver cirrhosis, or even liver failure with high mortality [10]. COVID-19, a kind of infectious respiratory illness, has increased incidence of EBV reactivation. EBV viremia was closely associated with COVID severity [11]. Chronic active EBV infection may result in progressive immunodeficiency, opportunistic infections, multiorgan failure, or even lymphomas [12].

On the other hand, primary EBV infection in children is likely to present with no symptoms [13]. However, the liver injury still merits attention, particularly in the infectious diseases. RTIs themselves are likely to occur frequently in children. Due to the potential harms of liver injury and possible influence of EBV infection in pediatric RTIs, to search for the easily available risk predictors for livery injury in pediatric RTIs is of great significance. Hence, early identification of liver injury indicators is helpful for pediatric health.

We conducted this retrospective study to analyze the incidence of EBV infection in different months, and the association between the clinical/laboratory parameters and liver injury in pediatric RTIs with EBV infection. We also performed the receiver operating curve and decision curve analysis to yield the predictive value of the clinical/laboratory parameters in the risk of liver injury in EBV infection. This in-depth investigation showed good clinical application value due to the fact that the clinical/ laboratory parameters are easily available in primary medical institutions, which promote the generalizability and operability of the early risk prediction of liver injury in pediatric RTIs with EBV infection.

## Materials and methods

### Subjects

We performed a retrospective analysis of EBV infection in children with RTIs. All the enrolled cases were the patients admitted to the Department of Pediatrics, Shanghai Sixth People’s Hospital, China. The study period was between January 2015 and December 2017. We enrolled the participants according to the following criteria: 1. children aged less than 14 years; 2. RTIs with co-infection of EBV; 3. the patients with systemic diseases that may affect the risk of EBV infection were excluded; 4. infectious mononucleosis cases were not enrolled; 5. cases with chronic liver injury diseases were not included. If the same case was enrolled, we chose the case with the most complete data. This study was performed in the retrospective style. All the collected participants information was de-identified. The data was analyzed anonymously.

### Data collection

We earnestly extracted the clinical and laboratory data from the electronical medical records. Two authors (Song Mao and Liangxia Wu) collected the data of age, gender, co-infected mycoplasma, influenza A/B, cytomegalovirus and birthweight (BW). In the meantime, we also collected the laboratory data, including white blood cell (WBC), c-reactive protein (CRP), platelet (PLT), erythrocyte sedimentation rate (ESR), procalcitonin (PCT), urine red blood cell (uRBC), blood urea nitrogen (BUN), serum creatinine (Scr), eosinophile granulocyte (EOS), calcium (Ca), phosphorus (P), and magnesium (Mg), and alanine aminotransferase (ALT). The authors verified the accuracy and quality of the data independently. The laboratory results extracted were the testing results within 3 days after admission to our hospital. The liver injury is defined as the value of ALT more than 90 U/L.

### Statistical analysis

The incidence of EBV infection in pediatric RTIs in terms of month was calculated. Variables of data of age/WBC/CRP/PLT/ ESR/PCT/uRBC/BUN/Scr/Ca/P/Mg /BW/EOS were expressed as means ± standard deviation (SD). Independent samples T test was applied to compare the differences of these parameters between EBV infection with and without liver injury. Chi square test was used to determine the difference of gender between EBV infection with and without liver injury. Univariate regression analysis was performed to determine the association between clinical/ laboratory parameters and the risk of liver injury in EBV infection. The yielded indexes were enrolled in the multivariate regression analysis. Decision curve analysis was performed to test the diagnostic value of WBC/PLT/BW in the liver injury of EBV infection. Receiver operating curve (ROC) analysis was performed to calculate the C-index of WBC in the liver injury of EBV infection. Smooth curve analysis was applied to determine the non-linear association between WBC and ALT. Threshold effect analysis was performed to yield the turning point of WBC in its association with ALT. All the analyses were performed by using R and EmpowerStats software. *P* < 0.05 was considered statistically significant, except where otherwise specified.

## Results

### Epidemiological findings

**Fig. 1:**
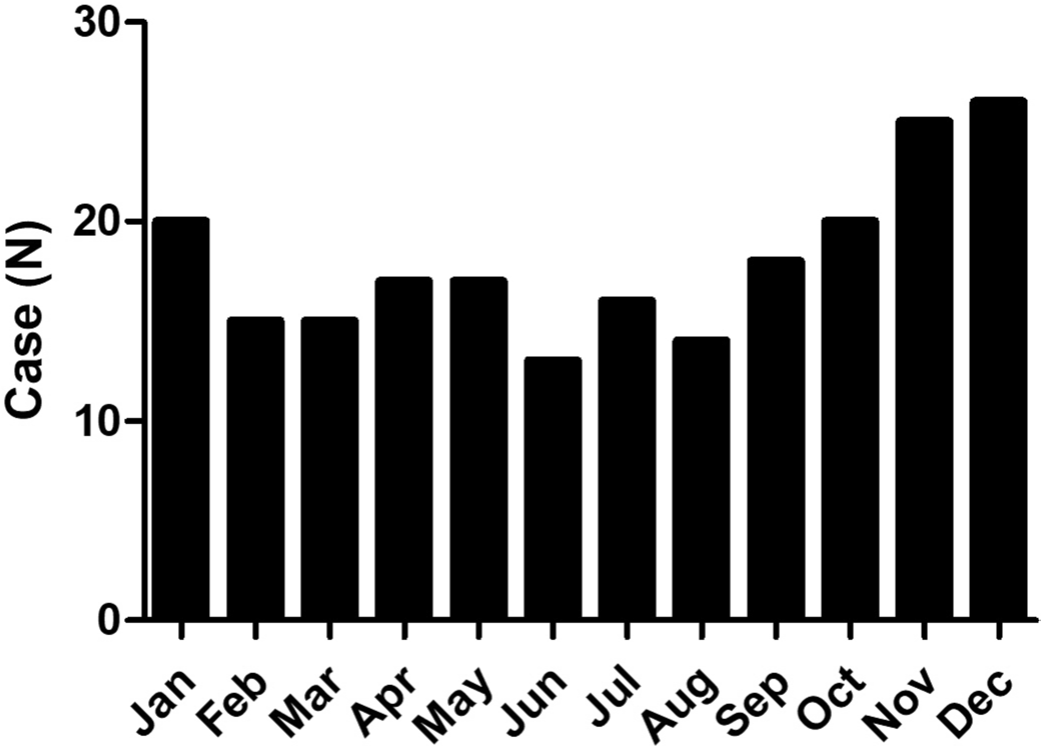
Distribution of EBV infection in pediatric RTIs in terms of month

A total of 216 pediatric RTIs with EBV infection were enrolled in our study. Among the recruited cases, 118 were male, 98 female, 36 with liver injury and 180 without liver injury. It was observed that 20; 15; 15; 17; 17; 13; 16; 14; 18; 20; 25 and 26 cases occurred in January; February; March; April; May; June; July; August; September; October; November; and December, respectively (Fig. 1). The clinical and laboratory parameters were presented in Table 1. Significant differences of WBC count and BW were observed between participants with and without liver injury (Table 1).

**Table 1:** Baseline characteristics of EBV infection in pediatric RTIs

Index	EBV infection without liver injury (180)	EBV infection with liver injury (36)	P
Age	4.4 ± 2.6	5.0 ± 3.3	0.674
Gender	0.8	1	0.797
WBC	9.1 ± 5.3	14.4 ± 6.3	< 10^–4^
CRP	28.2 ± 41.4	19.7 ± 28.7	0.935
PLT	255.6 ± 125.9	201.5 ± 57.4	0.079
ESR	23.9 ± 18.6	18.5 ± 12.3	0.364
PCT	0.5 ± 1.1	0.4 ± 0.6	0.089
uRBC	7.8 ± 8.3	8.1 ± 9.0	0.716
BUN	3.5 ± 2.7	3.1 ± 1.0	0.294
Scr	29.8 ± 7.1	32.4 ± 13.2	0.950
Ca	2.3 ± 0.1	2.3 ± 0.1	0.179
P	1.4 ± 0.3	1.4 ± 0.1	0.770
Mg	0.9 ± 0.1	1.0 ± 0.1	0.387
BW	3163.7 ± 1092.5	2559.7 ± 1265.0	0.006
EOS	0.2 ± 0.6	0.1 ± 0.1	0.579

Age (year), Gender (male/female ratio), WBC (10^9^/L), CRP (mg/L), PLT (10^9^/L), ESR (mm/hr), PCT (ng/ml), uRBC (n/ul), BUN (mmol/L), Scr (umol/L), Ca (mmol/L), P (mmol/L), Mg (mmol/L), BW (g), EOS (10^9^/L)

### Univariate regression analysis of the association between clinical/laboratory parameters and liver injury

WBC count and cytomegalovirus were significantly associated with liver injury in EBV infection (95% CI: 1.059–1.262, *p* = 0.001 and 95% CI: 2.020–21.967, *p* = 0.002, respectively, Table 2). No marked association between age, gender, CRP, PLT, ESR, PCT, uRBC, BUN, Scr, Ca, P, Mg, BW, EOS, co-infected influenza A/B and mycoplasma and liver injury in EBV infection was noted (Table 2).

**Table 2:** Univariate analysis of clinical and laboratory parameters in the liver injury risk in EBV infection

Index	β	95% CI	P
Age	0.083	0.908–1.300	0.367
Gender	-0.223	0.290–2.203	0.666
WBC	0.145	1.059–1.262	0.001
CRP	-0.007	0.970–1.016	0.559
PLT	-0.006	0.987–1.000	0.065
ESR	-0.020	0.947–1.014	0.243
PCT	-0.044	0.669–1.369	0.809
uRBC	0.004	0.946–1.065	0.892
BUN	-0.248	0.449–1.356	0.378
Scr	0.032	0.977–1.092	0.251
Ca	-3.481	0.000–4.594	0.173
P	-0.199	0.099–6.814	0.854
Mg	3.252	0.019–3.567	0.378
BW	0.000	0.999–1.000	0.053
EOS	-12.465	0.000–5.75	0.139
Virus	1.896	2.020–21.967	0.002
Influenza	0.248	0.249–6.602	0.767
Mycoplasma	0.630	0.667–5.284	0.232

Age (year), Gender (male/female ratio), WBC (10^9^/L), CRP (mg/L), PLT (10^9^/L), ESR (mm/hr), PCT (ng/ml), uRBC (n/ul), BUN (mmol/L), Scr (umol/L), Ca (mmol/L), P (mmol/L), Mg (mmol/L), BW (g), EOS (10^9^/L)

### riate regression analysis of the asMultivariate regression analysis of the association between WBC count, PLT, BW, and cytomegalovirus and liver injury

**Fig. 2:**
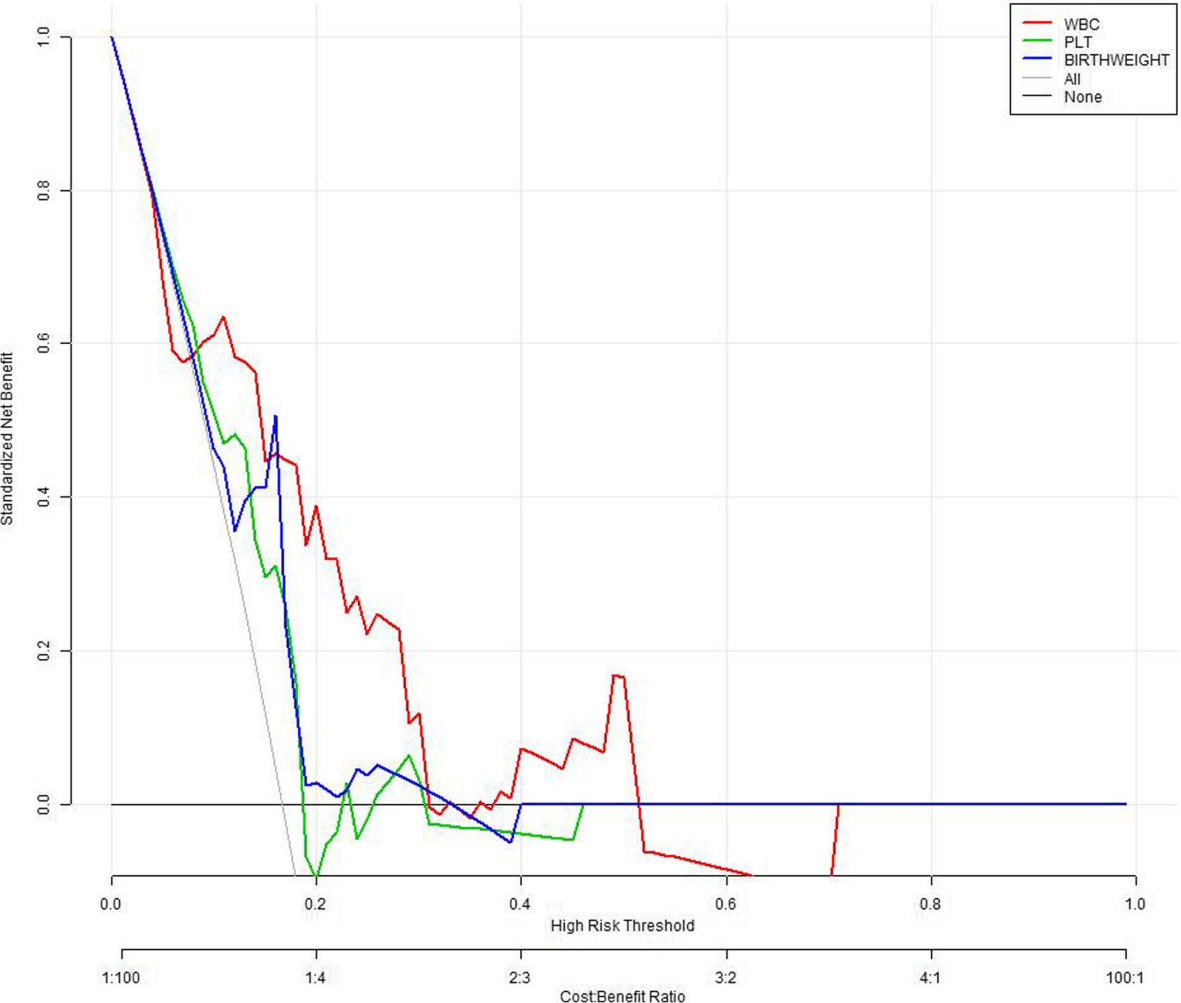
Decision curve analysis of risk prediction of liver injury in EBV infection

**Fig. 3:**
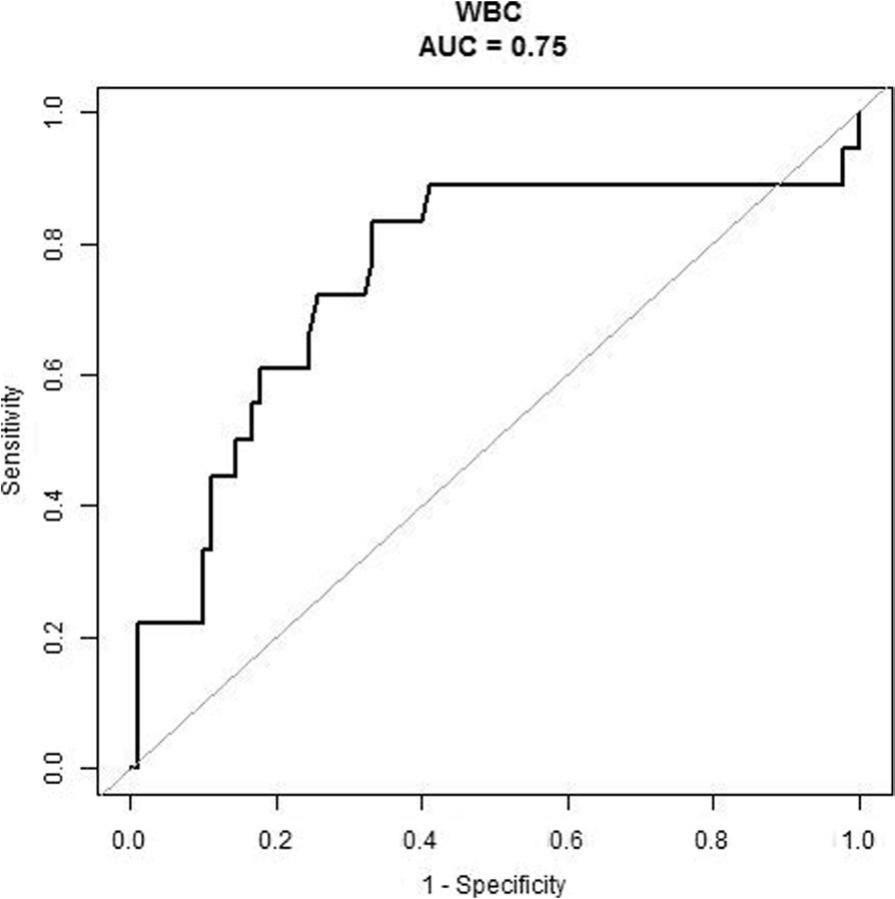
ROC analysis of the association between WBC count and liver injury

Cytomegalovirus infection was independently associated with liver injury in EBV infection (OR = 6.972, 95% CI = 1.648–29.490, *p* = 0.008, Table 3). Cytomegalovirus infection demonstrated 5.972 more times of liver injury risk. WBC count was independently associated with liver injury in EBV infection (OR = 1.169, 95% CI = 1.051–1.301, *p* = 0.004, Table 3). The P interaction value between WBC count and cytomegalovirus was 0.149. The decision curve analysis showed that WBC count had larger area under curve compared with PLT and BW (Fig. 2). ROC analysis yielded the c-index of WBC count: 0.75 and the cut-off point of 8.3 (Fig. 3).

**Table 3:** Multivariate analysis of clinical and laboratory parameters in the risk liver injury in EBV infection

Index	OR	95% CI	P
WBC	1.169	1.051–1.301	0.004
PLT	0.993	0.985–1.000	0.066
BW	0.999	0.999–1.000	0.945
Virus	6.972	1.648–29.490	0.008

Age (year), Gender (male/female ratio), WBC (10^9^/L), CRP (mg/L), PLT (10^9^/L), ESR (mm/hr), PCT (ng/ml), uRBC (n/ul), BUN (mmol/L), Scr (umol/L), Ca (mmol/L), P (mmol/L), Mg (mmol/L), BW (g), EOS (10^9^/L)

### A non-linear association between WBC count and ALT

**Fig. 4:**
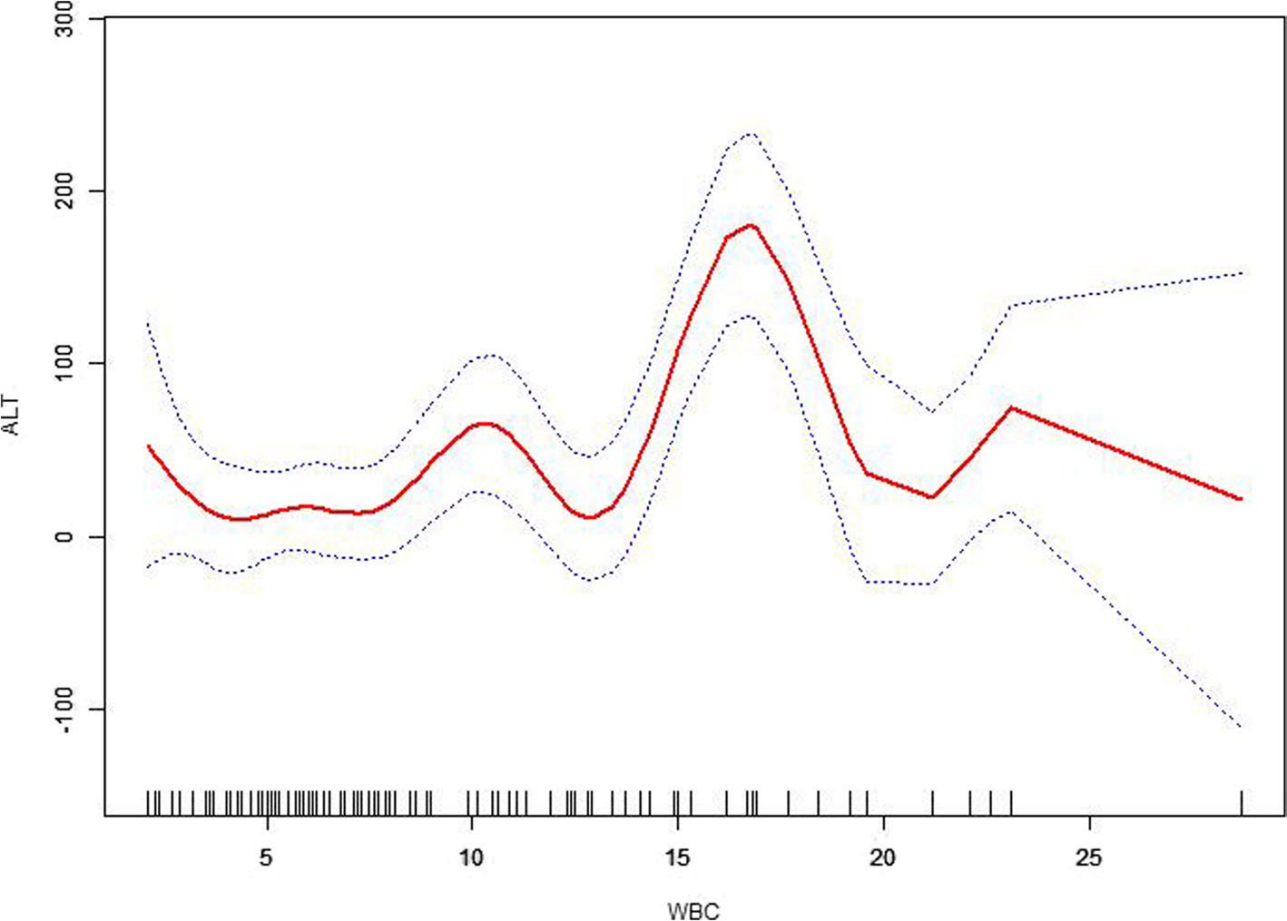
A non-linear association between WBC count and ALT

The turning point of WBC count in its association with ALT was 16.8 (Fig. 4). The *p* value before and after the turning point was < 0.001 and 0.123, respectively (Fig. 4).

## Disscussion

EBV, an ubiquitous virus, is likely to cause lifelong infection with a high incidence both in adult and children [14]. EBV can infect and activate B and T lymphocytes [15]. EBV infection is prone to occur in cases with lower immunity. Meanwhile, EBV infection affects the immune system, leading to the onset of certain immunological disorders, such as systemic lupus erythematosus, even cancers [16]. Liver injury was aggravated in EBV infection, meanwhile liver cirrhosis had high EBV infection rate [17]. The possible mutual role of EBV infection and liver injury indicates the importance of the early risk prediction of liver injury in EBV infection. Our study focused on the risk factors for liver injury of EBV infection in children with RTIs. We found that cytomegalovirus infection demonstrated 5.972 more times of liver injury risk. WBC count was an independent biomarker for liver injury before the turning point of 16.8.These findings indicated that early monitoring and prevention of cytomegalovirus infection and inflammation may be helpful for improving the liver injury of EBV infection in pediatric RTIs.

EBV transmits primarily via the close contact, such as saliva. For children with RTIs, they are infected primarily through the saliva [18], which increased the transmission of EBV infection.

It was reported that respiratory infection was the second most and most common disease caused by primary EBV infection and EBV reactivation in children, respectively [19]. Chronic EBV infection may also present with refractory chronic sinusitis [20]. EBV co-infection was also likely to occur in COVID-19 infection [21]. These above-mentioned evidence indicated that EBV infection is likely to occur in RTIs.

We found that the mean age of EBV infection cases was less than 6 years, which may be due to the facts that older children are likely to have higher immunity compared with younger cases [22]. The younger children are likely to present with RTIs due to the cross infection with the co-infection of EBV. On the other hand, primary EBV infection often occurs early in life [23]. We also observed that the incidence of EBV infection is highest during the winter season. RTIs are also likely to occur in the cold season, which may account for the higher incidence of EBV infection in winter [24]. 

Co-infected cytomegalovirus independently increased the risk of liver injury of EBV infection, which may be due to the facts that the liver is one of the target organs of cytomegalovirus infection [25]. Cytomegalovirus infection is also a major cause of birth defects including liver injury, hearing and visual loss [26]. On the other hand, cytomegalovirus infection-induced immunologic response may be involved in the risk of liver injury [27]. Notably, we observed that co-infected mycoplasma and influenza A/B did not affect the risk of liver injury in EBV infection. It may be due to the facts that liver is not the main target organ of mycoplasma and influenza A/B. Therefore, we may pay more attention to the cytomegalovirus antibody levels in the EBV infection, particularly in the liver injury cases.

Inflammation is involved in the onset of liver injury [28]. Liver dysfunction occurred frequently in cases of sepsis [29]. Inhibition of inflammation protected against the liver injury [29]. Hepatocyte FoxO1 deficiency protected from liver fibrosis through reducing inflammation [30]. WBC count is a classical biomarker of inflammation. WBC adhesion molecules were differently modulated by inflammation and T cell contact [31]. Our study showed that WBC count was independently associated with the risk of liver injury, which was consistent with the idea that inflammation plays an important role in the development of liver injury. Notably, we found that no interaction was found between WBC count and cytomegalovirus, which further verify the independent role of cytomegalovirus infection in the risk of liver injury. We also found obviously positive association between WBC count and ALT before the turning point of 16.8, which indicated that moderate inflammation increased the level of ALT, serious inflammation may not demonstrate increasing level of ALT. We guessed that the serious inflammation may induce the liver necrosis without ALT release. On the other hand, the majority of EBV co-infection cases show mild increase of ALT levels without jaundice. Even some cases showed no obvious symptoms. Therefore, it is of great implications to monitor the WBC count in EBV infection.

Our study has important clinical strengths that we clarified the independent association between WBC count/cytomegalovirus infection and liver injury risk, we also elucidated the non-linear relationship between WBC count and ALT. The liver injury induced by EBV infection is mainly mild and self-limiting, but a small number of cases presented with fulminant hepatic failure [32]. Early, convenient and low-cost way to identify the high-risk population of liver injury in EBV infection in children, particularly the young children is of great significance. The fingertip blood routine for WBC count is an easily feasible way for the young children without the need of vein blood drawing. Meanwhile, several limitations should be considered in our study. First, certain signal pathways may affect the role of EBV infection in liver injury. Hence, an in-depth study should be performed in the future. Second, the dynamic changes of biochemical indexes are helpful for the understanding the role of other factors in EBV infection in pediatric RTIs. Due to the lack of follow-up data, we were unable to conduct the analysis. Further studies should be performed to clarify this issue. Finally, the retrospective study design might produce recall bias, which may affect the results. A prospective study design was likely to result in a more detailed and accurate surveillance during the follow-up. Therefore, a long-term and prospective study design should be used by removing the confounding factors.

## Conclusions

Our investigation indicated that EBV infection is likely to occur in every month, particularly in the winter season. Cytomegalovirus infection demonstrated 5.972 more times of liver injury risk. WBC count was an independent biomarker for liver injury before the turning point of 16.8.

In terms of our findings, future studies should be performed to address these two issues (1) clarification of the detailed mechanisms of the interactions between other pathogens and EBV infection, (2) long-term and detailed follow-up of the alterations of various indexes and prognosis of EBV infection with a favorable study design. (3) the impact of various factors on the recovery of liver function.

We should pay more attention to the risk of EBV infection in the winter. Cytomegalovirus infection and WBC count merit attention in the monitoring of possible liver injury in EBV infection among pediatric RTIs.

## Data Availability

The datasets used and/or analyzed during the current study are available from the corresponding author on reasonable request.

## Abbreviations

EBV: Epstein-Barr virus
ESR: Erythrocyte sedimentation rate
RTIs: Respiratory tract infections
PCT: Procalcitonin
WBC: White blood cell
uRBC: Urine red blood cell
ALT: Alanine aminotransferase
BUN: Blood urea nitrogen
PLT: Platelet
Scr: Serum creatinine
BW: Birthweight
EOS: Eosinophile granulocyte
CRP: C-reactive protein
Ca: Calcium
P: Phosphorus
Mg: Magnesium
SD: Standard deviation
ROC: Receiver operating curve
